# Beyond cognitive distortions: the mediating role of passion between erroneous cognitions and gambling severity

**DOI:** 10.1080/00049530.2026.2726226

**Published:** 2026-09-07

**Authors:** Chris James Serafim, Ebony Sans, Adam Gunnoo

**Affiliations:** a Institute for Health and Sport, Victoria University, Melbourne, VIC, Australia; b School of Health and Biomedical Sciences, Royal Melbourne Institute of Technology, Melbourne, VIC, Australia

**Keywords:** Structural equation modelling, gambling, obsessive passion, harmonious passion, moderation

## Abstract

**Objective:**

While erroneous gambling cognitions and gambling passion (harmonious and obsessive) are recognised as key factors underpinning an individual’s gambling, limited research has examined whether passion explains how cognitive distortions relate to gambling severity.

**Method:**

Using structural equation modelling (SEM), authors evaluated the indirect paths of harmonious and obsessive passion between three key erroneous gambling cognitions among 630 gamblers aged between 18 and 73 years (M_age_ = 38.56, SD_age_ = 11.52).

**Results:**

Controlling for gambling preference, frequency, and social desirability, predictive control exhibited a significant negative indirect path to gambling severity via harmonious passion, whereas obsessive passion positively mediated the relationship between illusion of control, interpretive bias, and gambling severity. Furthermore, obsessive passion significantly (negatively) moderated the relationship between interpretive bias and gambling severity.

**Conclusion:**

Findings suggest that when individuals overestimate predictive control, they may cognitively minimise risks associated with their gambling. Additionally, internal pressure from obsessive passion could inflate perceived control over random outcomes and heighten the tendency to attribute outcomes to skill. Moderation results indicated obsessive passion may exert a stronger influence on gambling severity than interpretive bias. Overall, findings highlight the need to target both gambling cognitions and passion when addressing an individual’s gambling severity.

## KEY POINTS

What is already known about this topic:

1. Erroneous gambling cognitions (such as illusion of control and interpretive bias) are well-established cognitive distortions that directly contribute to the maintenance of gambling severity.
2. The Dualistic Model of Passion (harmonious and obsessive passion) is a recognised motivational framework that independently drives an individual’s gambling engagement and propensity.
3. While both cognitive and motivational factors are known predictors of gambling severity, limited research has examined their structural relationships or explored how underlying passion functions as a factor that can reinforce or mitigate the influence of these cognitive distortions.

What this topic adds:

1. Utilising structural equation modelling (SEM) of (*n* = 630) gamblers, this study demonstrates that harmonious and obsessive passion differentially mediate the pathways between specific erroneous cognitions and gambling severity.
2. Controlling for harmonious passion, obsessive passion significantly and negatively moderated the relationship between interpretive bias and gambling severity, indicating that intense motivational drives can become the dominant force propelling severity.
3. Findings indicate that clinical interventions should expand beyond purely cognitive restructuring to holistically target the underlying motivations that drive an individual’s gambling.

## Introduction

Gambling is a popular activity currently legal in over 80% (156) of countries worldwide (Ukhova et al., 2024). While many individuals gamble without disruption to social functioning (Raybould et al., 2021; Welte et al., 2015), some experience significant negative consequences (Raybould et al., 2021), typically as a result of problem gambling. Problem gambling can be defined as any form of gambling that leads to harm, distress, and/or negatively impacts an individual’s life pursuits (Ferris & Wynne, 2001; Williams & Volberg, 2013). Current estimates suggest that the global prevalence of problem gambling ranges from 1.29% to 1.41% (Gabellini et al., 2023; Tran et al., 2024), with this behaviour often linked with several intra- and interpersonal harms, such as depression and anxiety symptomatology (Cardwell et al., 2022; N. A. Dowling et al., 2019; Ozoliņa et al., 2025), family violence (N. Dowling et al., 2016; Suomi et al., 2019), and homelessness (see Vandenberg et al., 2022). Given the adverse outcomes, researchers have sought to identify underlying characteristics and risk factors of problem gambling, with the manifestation of erroneous gambling cognitions outlined as a key predisposing and perpetuating factor of this behaviour.

## Erroneous gambling cognitions

Erroneous gambling cognitions (i.e., “distorted” cognitions) are recognised as the limited consideration of the random nature of gambling, leading to the idea that an individual can control and/or predict gambling outcomes (Delfabbro et al., 2006; Ladouceur, 2004). Common erroneous gambling cognitions noted within the literature include *illusion of control* (i.e., the false belief that an uncontrollable event can be controlled), *interpretive bias* (i.e., relating gambling outcomes to an individual’s level of skill), and *predictive control* (i.e., the belief that an individual can foresee and determine gambling outcomes) (Raylu & Oei, 2004). While erroneous gambling cognitions are common across the gambling spectrum (Krébesz et al., 2023), problem gamblers are often noted to be more likely to endorse and experience erroneous gambling cognitions than non-problem and low-risk gamblers (Ciccarelli et al., 2016; Joukhador et al., 2003; Michalczuk et al., 2011). Some researchers have also argued that specific types of cognitive distortions can distinguish problem gamblers from non-gamblers and low/at-risk gamblers (see Labrador et al., 2020). Accordingly, empirical evidence suggests that past experiences of erroneous gambling cognitions predict both current and future problem gambling behaviours (Leonard et al., 2021; Nicholson et al., 2016; Philander & Gainsbury, 2023; Schulte et al., 2020; Yakovenko et al., 2016). However, the bi-directionality surrounding this relationship remains equivocal. For example, Nicholson et al. (2016) suggested that problem gambling is associated with future erroneous cognitions, whilst Yakovenko et al. (2016) argued that cognitive distortions occur prior to an individual’s gambling behaviour. Thus, these findings may indicate the role of erroneous cognitions in predicting gambling behaviour, and how engaging with gambling may reinforce said cognitions. Nevertheless, while erroneous gambling cognitions are well-established predictors of problem gambling, research also indicates that specific motivational factors, particularly passion, play an important role in the exacerbation of this behaviour (Morvannou et al., 2017, 2018; Philippe & Vallerand, 2007; Ratelle et al., 2004; Serafim, 2026; Skitch & Hodgins, 2005).

## Gambling passion

Passion is often defined as the strong inclination towards an activity that an individual finds important and invests time and energy in (Vallerand, 2010; Vallerand et al., 2003). One of the popular conceptualisations of passion is the motivational Dualistic Model of Passion (DMP; Vallerand et al., 2003), which details passion as a dual-construct comprising *harmonious* and *obsessive* passion (Rousseau et al., 2002; Vallerand, 2010; Vallerand et al., 2003). Harmonious passion is recognised by engagement in enjoyable, pleasurable activities while maintaining balance in other aspects of life (Vallerand et al., 2003). However, whilst harmonious passion may be linked to subjective wellbeing (Oikonomidis et al., 2019), individuals may still experience difficulty in managing their gambling behaviour. Indeed, evidence indicates that harmonious passion is also associated with higher levels of gambling severity (Morvannou et al., 2017, 2018; Serafim, 2026).

Conversely, obsessive passion is recognised as the internal pressure to engage in an activity that may result in internal conflict or adverse consequences (Vallerand et al., 2003) and is frequently linked with increased gambling severity (Morvannou et al., 2017, 2018; Philippe & Vallerand, 2007; Serafim, 2026). Researchers have also argued that obsessive passion may discriminate problem gamblers from social/recreational gamblers (Back et al., 2011; Philippe & Vallerand, 2007; Serafim, 2026). Interestingly, while problem gamblers are known to report high levels of obsessive passion, multiple studies have demonstrated that problem gamblers score higher in harmonious passion (Castelda et al., 2007; Morvannou et al., 2017, 2018; Skitch & Hodgins, 2005), with few showing the inverse (Philippe & Vallerand, 2007; Ratelle et al., 2004; Serafim, 2026). Demonstrably, rather than operating as opposing constructs, obsessive and harmonious passion represent unique constructs that can co-occur and influence one’s gambling severity simultaneously, potentially through erroneous gambling cognitions.

## Gambling passion and erroneous gambling cognitions

Although harmonious and obsessive passion are established as key gambling motives, how these internalisations interact with other factors of this behaviour, such as erroneous gambling cognitions, has received little attention. Consequently, the specific relationship between passion and erroneous gambling cognitions remains mostly unexplored. To the best of the authors’ knowledge, only Y. Hu and Cai (2025) have examined the interaction between passion and erroneous gambling cognitions, finding that harmonious and obsessive passion mediated the relationship between illusion of control and problem gambling severity in a sample of Chinese lottery gamblers. However, Y. Hu and Cai (2025) examined only the illusion of control, restricting the scope of findings and neglecting interactions between passion and other erroneous cognitions (e.g., interpretive bias and predictive control) in relation to gambling severity. Nevertheless, these findings suggest that emotional intensity associated with gambling passion may compromise objective reasoning and increase the predictive validity and control an individual perceives to have over gambling outcomes. However, few studies have examined whether gambling passion operates as an underlying factor linking erroneous cognitions to heightened gambling severity.

## The current study

Whilst passion and gambling cognitions represent distinct psychological constructs, emerging evidence suggests that both play meaningful roles in shaping an individual’s gambling engagement (Y. Hu & Cai, 2025). Specifically, an individual’s erroneous cognitive distortions may be channelled through their passion for gambling, which in turn may contribute to higher levels of gambling. Although passion for gambling may reflect broader cognitive deficits (Serafim, 2026), little research has examined whether passion helps explain the relationship between erroneous gambling cognitions and gambling propensity. As both passion and erroneous cognitions are established predictors of gambling severity, analysing their structural relationships may yield a more nuanced understanding of how motivational factors reinforce or mitigate the influence of erroneous beliefs on an individual’s behaviour and subsequent severity. As such, the current study investigated the mediating roles of obsessive and harmonious passion between erroneous gambling cognitions and gambling severity. The following hypotheses were proposed:

H1: *All examined erroneous gambling cognitions will positively predict high levels of gambling severity.*

H2: *Harmonious passion will negatively predict gambling severity, while obsessive passion will positively predict gambling severity.*

H3: *All examined erroneous gambling cognitions will positively predict obsessive and harmonious passion.*

H4: *Harmonious and obsessive passion will significantly mediate the relationship between all examined erroneous gambling cognitions and gambling severity.*

**Figure 1.**
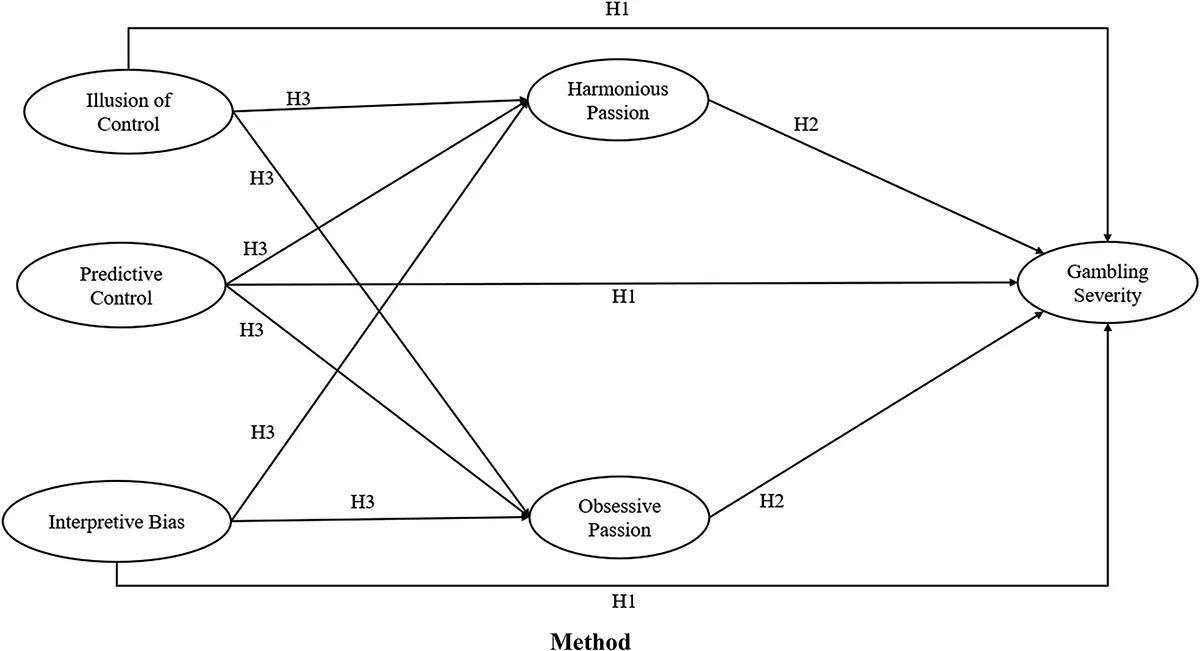
Hypothesised model of erroneous gambling cognitions, passion, and gambling severity.

Figure 1 represents the hypothesised model under investigation, depicting the conceptual link between the erroneous gambling cognitions within the GRCS, obsessive and harmonious passion and gambling severity.

## Method

### Participants

A total international sample of 844 participant responses was collected, with 738 (87.4%) from Prolific.co and 106 (12.6%) from the author’s local (Australian) community. All participants were required to be over the age of 18 and have gambled at least once in the past week. After the data were collected, a secondary inclusion criterion was applied. Participants who did not complete > 90% of the survey or did not correctly answer two of the three attention check questions were excluded. A CAPTCHA was also added to the online survey to detect whether participants used bots, and no evidence of bot use was found.

It should also be noted that a total of six missing values were found; however, none exceeded the 5% *missing completely at random* (MCAR) threshold. Also, as no systematic differences or predictors were found for the missing data, and the *p*-value for the *expectation maximisation* (EM) was noted to be > .05 (Little’s MCAR test: *χ*^*2*^ (930) = 824.51, *p* = .994), the EM method was deemed appropriate and was used to account for the missing data.

After excluding 214 participants, a total of 630 gamblers (*n* = 92 from the general community, *n* = 538 crowdsourced from Prolific) aged between 18 and 73 years (*M*_age_ = 38.56, *SD*_age_ = 11.52) were retained for analysis. Although some researchers contend that paid convenience samples may introduce bias into responses (Pickering & Blaszczynski, 2021), others argue that paid online convenience sampling enables the collection of larger samples from targeted sub-groups (Russell et al., 2022). Furthermore, the anonymity afforded by online surveys may promote more candid responses (Russell et al., 2022). Scholars have also indicated that online gambling surveys are a reliable method for obtaining representative samples of gamblers for research (Heirene et al., 2025). The mean level of gambling severity, based on the scoring criteria from the *Problem Gambling Severity Index* (PGSI; Ferris & Wynne, 2001), was 7.02 (SD = 6.07), suggesting that participants in the current sample were at moderate risk of problem gambling. The remaining demographic and clinical information for the current sample are presented in Table 1.

**Table 1.** Demographic and clinical Information (*n* = 630).

Demographic information	
**Age, *M* (*SD*)**	38.56 (11.52)
**Gender, n (%)**	
Male	457 (72.6%)
Female	169 (26.6%)
Non-Binary	2 (.4%)
Transgender Male	2 (.4%)
Transgender Female	–
Prefer Not to Answer	–
**Relationship Status, n (%)**	
Married/Defacto	233 (37.0%)
In a Relationship (Living together)	137 (21.7%)
In a Relationship (Living separately)	81 (12.9%)
Separated/Divorced	21 (3.3%)
Single	152 (24.1%)
Widowed	6 (1.0%)
**Education, n (%)**	
Partially Completed High School	6 (1.0%)
Full High School Completion	102 (16.2%)
Vocational Education/Trade (TAFE)	82 (13.0%)
Partially Completed University	45 (7.1%)
Full University Completion	95 (15.1%)
Completed Undergraduate	177 (28.1%)
Completed Postgraduate	123 (19.5%)
**Employment Status, n (%)**	
Employed (Full Time)	467 (74.1%)
Employed (Part Time)	82 (13.0%)
Employed (Casual)	20(3.2%)
Unemployed (not looking for work)	26 (4.1%)
Unemployed (looking for work)	21 (3.3%)
Retired	14 (2.2%)
**Income (AUD), n (%)**	
$0 to $30,000	102 (16.2%)
$30,001 to $60,000	184 (29.2%)
$60,001 to $90,000	202 (32.1%)
$90,000 to $120,000	91 (14.4%)
$120,000 plus	51 (8.1%)
**Geographical Location, n (%)**	
Inner City	128 (20.3%)
Inner Suburbs	170 (27.0%)
Outer Suburbs	173 (27.5%)
Regional City	70 (11.1%)
Rural	68 (14.0%)
Remote	1 (0.2%)
**Gambling Frequency, n (%)**	
Once per week	178 (28.3%)
Two to six times per week	303 (48.1%)
Daily	110 (17.5%)
Multiple times per day	39 (6.2%)
**Clinical Information**	***M*** **(*SD*)**
**Gambling Severity - (PGSI)**	7.02 (6.07)
**Harmonious Passion - (GPS)**	3.10 (1.50)
**Obsessive Passion – (GPS)**	2.66 (1.75)
**Illusion of Control - (GRCS)**	2.32 (1.45)
**Predictive Control – (GRCS)**	3.32 (1.46)
**Interpretive Bias – (GRCS)**	3.91 (1.57)
**Gambling Frequency**	5.01 (.840)
**Strategic Gambling**	2.95 (1.21)
**Nonstrategic Gambling**	3.09 (1.29)
**Social Desirability – (S-MCSDS)**	2.30 (1.48)

Note: *M =* Mean, *SD =* Standard Deviation, *n =* Sample Size, PGSI = Problem Gambling Severity Index, GPS = Gambling Passion Scale, GRCS = Gambling Related Cognition Scale, Short-Marlowe-Crowne Social Desirability Scale (S-MCSDS).

## Materials

### Gambling preference

The authors selected various gambling activities based on an *a priori* literature review. Items were examined on an 8-point Likert scale ranging from 1 (not at all) to 8 (daily), with higher scores indicating greater gambling frequency. A total of 22 items (20 multiple-choice and 2 short-answer) were included, with the following prompt: *“Indicate which of the following types of gambling you have done in the past 12 months*” presented for each item. The two short-answer questions were optional. For the purpose of the current study, only 15 items were selected, which pertained to electronic gambling machines (EGM), gambling on card games (e.g., poker, baccarat and blackjack), gambling on table games (e.g., roulette, craps and backgammon), gambling on the lottery, animals (i.e., horses and dogs), sports, e-sports and fantasy sports/e-sports. Items surrounding e-sports gambling were included to ensure accurate representation of emerging and/or novel gambling activities. These gambling activities were divided into two gambling modalities: “strategic” (i.e., gambling on card games, animals, sports, e-sports, and fantasy sports/e-sports), and “non-strategic” (i.e., EGM’s, gambling on table games, and the lottery). Given the structure of the survey item, backgammon could not be separated from the accompanying non-strategic activities (i.e., roulette and craps). While backgammon inherently involves strategic elements, the overall item was categorised as non-strategic to reflect the majority of activities measured within that specific item.

#### Gambling Related Cognition Scale (GRCS)

The GRCS (Raylu & Oei, 2004) is a 23-item self-report questionnaire that assesses cognitive-related gambling distortions measured on a 7-point Likert scale ranging from 1 (*strongly disagree*) to 7 (*strongly agree*). The GRCS assesses cognitive-related gambling distortions across five dimensions, including: *gambling expectancies* (i.e., positive views an individual has towards the benefits of gambling), *illusion of control* (i.e., the extent to which an individual believes that they can control the outcome of their gambling), *predictive control* (i.e., the belief that an individual can predict the result of their gambling), *inability to stop gambling* (i.e., the view that one cannot stop gambling due to the urges they have for their gambling) and *interpretive bias* (i.e., tendency to interpret and/or attribute gambling outcomes to ones level of skill). For the current study, the GRCS dimensions of gambling expectancies and inability to stop gambling were excluded. This occurred as gambling expectancies and the perceived inability to stop gambling are argued to be more closely related to one’s pervasive interpersonal beliefs adopted from substance-use disorders research (Lee et al., 1999) as opposed to the erroneous cognitions/biases an individual holds towards their gambling. A similar approach in excluding these two dimensions has also been adopted in previous research (see Bonnaire et al., 2022; Whelan et al., 2021). Example questions for the GRCS include: “*Relating my losses to bad luck and bad circumstances makes me continue gambling*” and *“I collect specific objects that help increase my chances of winning*”. For this study, Cronbach’s α for the utilised GRCS sub-scales ranged from good to excellent (α_illusion of control_ = .88, α_predictive control_ = .94, α_interpretive bias_ = .85). McDonald’s ω for each sub-scale of the GRCS was also good (ω_illusion of control_ = .88, ω_predictive control_ = .94, ω_interpretive bias_ = .85).

#### Gambling Passion Scale (GPS)

The GPS (Rousseau et al., 2002) is a 14-item self-report questionnaire assessing gambling passion. The GPS measures passion on a 7-point Likert scale ranging from 1 (*not agree at all*) to 7 (*very strongly agree*). Five items within the GPS assess harmonious passion, for example, “*Playing this gambling game is in harmony with the other activities in my life*”. Five items assess obsessive passion, for example, “*I have almost an obsessive feeling for this gambling game*”. High harmonious passion scores indicate that an individual perceives their gambling as a controllable and autonomous behaviour, while high obsessive passion scores reflect a rigid, uncontrollable urge to gamble. The remaining four items are considered general criteria for passion, for example, “*This gambling game is important for me*”, and were not examined in this study. For the current study, Cronbach’s α for the two sub-scales within the GPS was excellent (α_harmonious_ = .92, α_obsessive_ = .96). McDonald’s ω for each sub-scale of the GPS was also good (ω_harmonious_ = .92, ω_obsessive_ = .96). The internal consistency of the full scale of the GPS was excellent and good for Cronbach’s α and McDonald’s ω, respectively (Cronbach’s α = .95; McDonald’s ω = .95).

#### Problem Gambling Severity Index (PGSI)

The PGSI (Ferris & Wynne, 2001) is a brief 9-item questionnaire that measures problem gambling via a 4-point Likert scale ranging from 0 (*never*) to 3 (*almost always*). Items within the PGSI include “Have you felt guilty about the way you gamble or what happens when you gamble?” Scores for each item within the PGSI were summed together to create a total gambling severity score for each participant. For this study, the internal consistency of the PGSI was excellent and good for Cronbach’s α and McDonald’s ω, respectively (Cronbach’s α = .93; McDonald’s ω = .93).

#### Short-Marlowe-Crowne Social Desirability Scale (S-MCSDS)

The S-MCSDS (Reynolds, 1982) was included to assess potential response bias. All five included items were dichotomously scored as “True” or “False”. True (i.e., correct) responses were coded as 0, while false (i.e., incorrect) responses were coded as 1. Responses were then summed to calculate total scores, resulting in a score ranging from 0 (low social desirability) to 5 (high social desirability). No items made any specific reference to gambling. Example items from the S-MCSDS include “*There have been occasions when I took advantage of someone*”.

### Covariates

Given that gambling frequency and preference are reportedly associated with gambling severity (Gainsbury et al., 2019; Mathieu et al., 2020; Ronzitti et al., 2016), these variables were considered as potential control variables. Social desirability was also considered as a covariate, as socially desirable response patterns have been known to occur in online samples (Bäckström & Björklund, 2013; Fang et al., 2016; Latkin et al., 2017; Pickering & Blaszczynski, 2021; Ried et al., 2022; Schell et al., 2021). As gambling frequency, gambling preference (i.e., strategic and non-strategic), and social desirability were significantly correlated with most primary study variables (see Table 3), all were included as control variables in the SEM. Mean composite scores were computed for social desirability, strategic gambling, and non-strategic gambling. A mean composite score was also computed for gambling frequency, measured on a 7-point Likert scale, ranging from 1 (<1 per month) to 7 (multiple times per day). Presenting these variables parsimoniously prevented unnecessary model complexity and preserved statistical power for our core latent structural paths.

### Procedure

Following ethics approval (ethics ID: HRE25-043), participants completed an online survey, facilitated via *Qualtrics*. Participants needed to check the box at the bottom of the landing page, indicating that consent had been given. All participants provided digital consent. This survey took approximately 15 to 20 minutes to complete, as survey items within this study were part of a larger questionnaire focused on gambling. Upon completing the study, the participants recruited via Prolific.co received $10 pro rata for their time. Participants recruited via the general community were not paid. The advertisement flyers and consent forms used for this study are available on Open Science Framework (OSF) (see https://osf.io/7x6ca).

### Statistical analysis

Data were analysed using SPSS and *R-Studio* (version 4.3.3) (The Posit Team, 2026), using the “lavaan” package for Structural Equation Modelling (SEM) (Rosseel, 2012). Correlations between variables were assessed using Pearson’s correlation coefficient (*r*). The alpha level was set at .05 for all analyses within this study. Maximum likelihood estimation was applied with an alpha level of .05. Total, direct, and indirect (mediating) effects were evaluated using 10,000 bootstrap resamples (95% CI). Model fit was evaluated against established benchmarks (Browne & Cudeck, 1989; L. Hu & Bentler, 1999; Kline, 2023): (χ^2^/*df*) <5, *Root Mean Square Error of Approximation* (RMSEA < .08), *Standardised Root Mean Square Residual* (SRMR < .1), and *Comparative fit index* (CFI), *Tucker–Lewis index* (TLI), *Goodness-Of-Fit Index* (GFI) and *Adjusted Goodness-Of-Fit Index* (AGFI) >.85. Incremental variance (Δ*R*^2^) for the main predictors was determined via nested parameter constraint tests using (*β* = 0), which were conducted and compared against the full model.

Preliminary collinearity diagnostics were conducted across all study variables, with substantial overlap noted between inability to stop gambling and obsessive passion (*r* = .858). Both variables also exceeded the Variance Inflation Factor (VIF) threshold of >5.0 (*inability to stop gambling* = 5.31; obsessive passion = 5.35), while nearing the minimum Tolerance threshold of <.20 (*inability to stop gambling* = .188; *obsessive passion* = .187). Including the *inability to stop gambling* subscale eliminated multicollinearity, reducing obsessive passion’s VIF to 1.81, Tolerance = .553. Together with theoretical considerations, this justified excluding both the *inability to stop gambling* and *gambling expectancies* subscales of the GRCS. Final diagnostics confirmed no remaining multicollinearity issues (Tolerance > .20, min = .326; VIF < 5.0, max = 3.07). Full assumption testing, zero-order correlations, comparative collinearity diagnostics, data, and R-Studio execution syntax are publicly available on OSF (see https://osf.io/7x6ca).

Within the SEM, illusion of control, predictive control, and interpretive bias were entered as predictor variables, gambling severity as the outcome variable, and both passion constructs were entered as mediators. Social desirability, gambling frequency and gambling preference (i.e., strategic and non-strategic) were also added as control variables. To account for the theoretical overlap, harmonious and obsessive passion were entered as parallel mediators in the SEM, and their residual variances were allowed to covary. This approach enables estimation of the unique indirect effect associated with each mediator while controlling for their shared variance.

Multi-group SEM across discrete PGSI risk categories (i.e., non-problem, low-risk, moderate-risk, and problem gambling) was considered but deemed methodologically unviable due to zero variance (σ^2^ = 0). Given that an individual must score 0 on the PGSI to be classified as a non-problem gambler, the subgroup variance is zero, yielding a singular covariance matrix that prevents model convergence. While collapsing non-problem and low-risk groups could circumvent this, doing so would artificially inflate variance and likely obscure the uniqueness of these distinct groups. Thus, retaining PGSI scores as a continuous variable preserved full sample variance without requiring the researchers to artificially collapse gambling groups.

## Results

### Correlation analysis

The zero-order and partial correlations controlling for gambling frequency, social desirability, and strategic /non-strategic gambling for all variables included in this study can be seen in Table 2. Zero-order correlations revealed that gambling severity correlated positively with illusion of control, predictive control, harmonious passion, gambling frequency, and strategic/non-strategic gambling (all weak), as well as obsessive passion and interpretive bias (moderately strong), with a weak negative correlation found for social desirability.

**Table 2.** Zero-order and partial correlations.

	Zero-order correlations										Partial correlations					
Variable	1	2	3	4	5	6	7	8	9	10	1	2	3	4	5	6
1. PGSI – Gambling Severity	–										–					
2. GRCS – Illusion of Control	.317***	–									.214***	–				
3. GRCS – Predictive Control	.262***	.650***	–								.172***	.645***	–			
4. GRCS – Interpretive Bias	.465***	.443***	.688***	–							.371***	.420***	.646***	–		
5. GPS – Harmonious Passion	.224***	.431***	.611***	.554***	–						.073	.364***	.543***	.470***	–	
6. GPS - Obsessive Passion	.679***	.364***	.464***	.598***	.493***	–					.622***	.342***	.413***	.522***	.425***	–
7. Social Desirability	−.232***	.371	−.188***	−.319***	−.162***	−.292***	–									
8. Gambling Frequency	.287***	.533	.227	.171***	.147***	.292***	−.130***	–								
9. Strategic Gambling	.330***	.350***	.317***	.302***	.434***	.245***	−.059	.201***	–							
10. Non-Strategic Gambling	.323***	.368***	.120**	.119**	.187***	.132***	.058	.135***	.595***	–						

Note: *n = 630, M* = Mean, *SD* = Standard Deviation, PGSI = Problem Gambling Severity Index, GRCS = Gambling Related Cognitions Scale, GPS = Gambling Passion Scale. Partial correlations for GPS and GRCS variables for controlled gambling frequency, social desirability, strategic and non-strategic gambling. Significant parameters are highlighted in bold text.***p < .001, **p < .01, *p < 0.05.

Illusion of control, predictive control, and interpretive bias demonstrated moderate positive correlations with both passion dimensions, weak positive associations with strategic/non-strategic gambling, and weak negative associations with social desirability. Interpretive bias also showed a weak positive correlation with gambling frequency. Finally, a weak positive correlation was found between harmonious and obsessive passion, with both forms of passion showing weak negative correlations with social desirability and weak positive correlations with gambling frequency, strategic and non-strategic gambling. This weak correlation between obsessive and harmonious passion justified specifying both passion constructs in parallel in the SEM, while allowing their residual variances to covary. When controlling for gambling frequency, strategic gambling, non-strategic gambling and social desirability, partial correlations mostly mirrored zero-order correlations across all variables, with the sole exception that the association between harmonious passion and gambling severity became non-significant.

### Structural equation model (SEM)

As chi-squared tests [χ^2^(600) = 2311.96, *p* < .001] can be biased due to variations in sample size, the corrected measure was used. As the degrees of freedom (χ^2^/df = 3.85) were less than 5, the model was seen to have a good overall fit. The fit indices for the current model were also noted to fall within acceptable ranges: RMSEA = .067, 90% *CI* [.064, .70], SRMR = .094, CFI = .90, TLI = .89. However, GFI = .80 and AGFI = .77 were slightly below the acceptable thresholds. Nevertheless, the overall model was recognised to have an acceptable fit.

**Figure 2.**
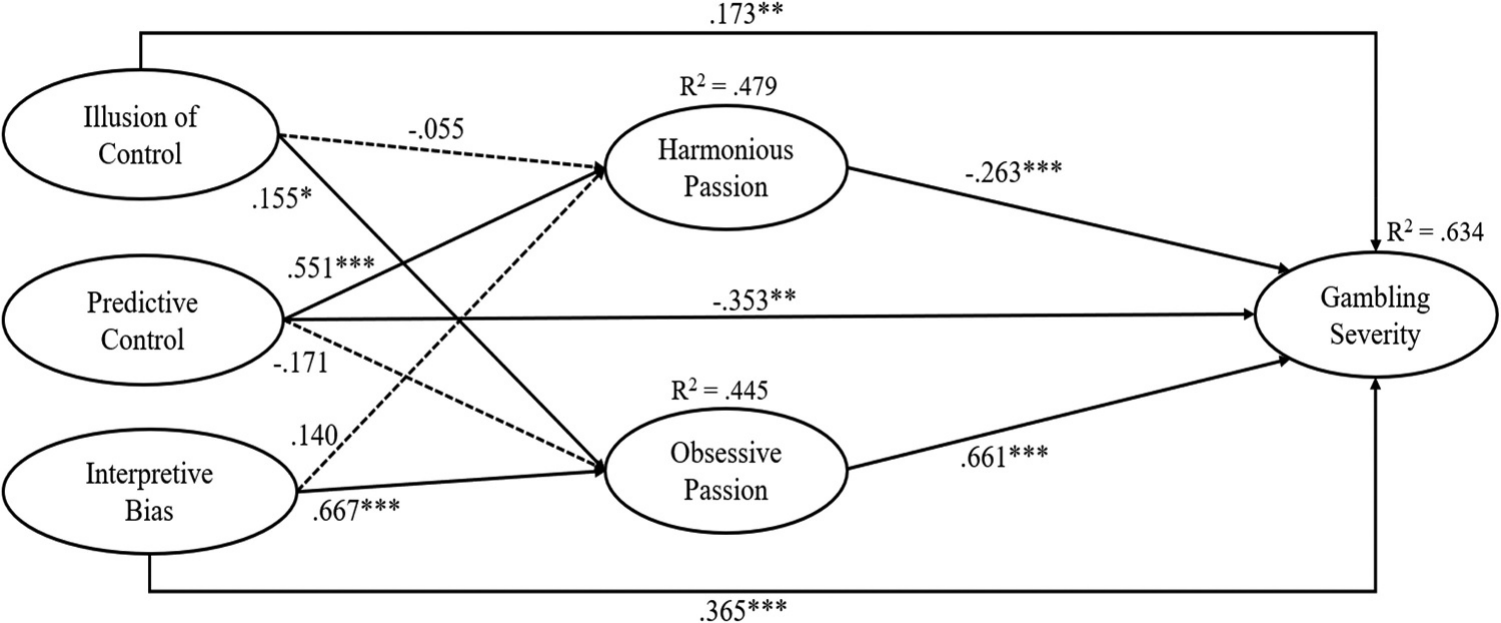
SEM with Standardised regression paths and coefficients.

Note: *n* = 630. ML estimation with bias-corrected bootstrapping (10,000 resamples) was employed. *β* = Standardised Beta. Simple arrows: significant path. Dotted arrows: non-significant path coefficients. The SEM controlled for the shared variance between passion constructs by including the alternative form of passion as a covariate when examining the other. SEM also controlled for gambling frequency, social desirability, and strategic and non-strategic gambling. *p < .05; ** *p* < .01; *** *p* < .001.

Figure 2 highlights the SEM and direct regression paths. A significant positive direct effect was found between illusion of control (*β* = .173, *p* = .003, 95% *CI* [.056, .290]) and interpretive bias on gambling severity (*β* = .365, *p* < .001, 95% *CI* [.179, .552]). Conversely, a significant negative direct effect was found between predictive control and gambling severity (*β* = −.353, *p* < .01, 95% *CI* [−.572, −.134]). Harmonious passion was also a significant negative predictor of gambling severity (*β* = −.263, *p* < .001, 95% *CI* [−.358, −.167]), whilst obsessive passion was a significant positive predictor (*β* = .661, *p* < .001, 95% *CI* [.562, .760]). A significant positive predictive effect was also found between predictive control and harmonious passion (*β* = .551, *p* < .001, 95% *CI* [.330, .772]), with interpretive bias (*β* = .667, *p* < .001, 95% *CI* [.491, .842]) and illusion of control (*β* = .155, *p* = .048, 95% *CI* [.001, .310]) revealed as significant positive predictors of obsessive passion.

For the control variables, strategic (*β* = .138, *p* < .01, 95% *CI* [.051, .224]) and non-strategic gambling (*β* = .177, *p* < .001, 95% *CI* [.101, .254]) were significant positive predictors of gambling severity, whilst strategic gambling was a significant positive predictor of harmonious passion (*β* = .305, *p* = .001, 95% *CI* [.211, .400]). With this, social desirability was a significant negative predictor of obsessive passion (*β* = −.107, *p* = .004, 95% *CI* [−.179, −.034]), while gambling frequency was a positive predictor of obsessive passion (*β* = .204, *p* < .001, 95% *CI* [.130, .278]). Overall, the model accounted for 63.4% of the variance in gambling severity (*R*^2^ = .634). Furthermore, the examined erroneous cognitions explained 45.5% of the variance in obsessive passion (*R*^2^ = .455) and 47.9% of the variance in harmonious passion (*R*^2^ = .479).

The overall model predicted 63.4% of variance (R^2^ = .634) with no incremental increase once harmonious passion was entered (Δ*R*^2^ = 0.0; 0.0%, *p* < .001). Conversely, constraining obsessive passion resulted in substantial variance inflation (Δ*R*^2^ = −.176; 17.6%, *p* < .001, *R*^2^_constrained_ = .810; 81.0%). Interpretive bias accounted for an additional 3.0% of unique variance (Δ*R*^2^ = .030; *p* < .001), predictive control accounted for 2.2% of unique variance (Δ*R*^2^ = .022; *p* < .001), with illusion of control accounting for 1.3% of unique variance (Δ*R*^2^ = .013; *p* < .001).

The indirect effects (when controlling for gambling frequency, social desirability, and strategic and non-strategic gambling) are summarised in Table 3. Results revealed a significant negative indirect effect between predictive control (*β* = −.145, *p* < .001, 95% *CI* [−.216, −.073]), mediated by harmonious passion, on gambling severity. A significant, positive indirect effect was also noted between illusion of control (*β* = .102, *p* = .049, 95% *CI* [.001, .204]), mediated by obsessive passion, and interpretive bias (*β* = .441, *p* < .001, 95% *CI* [.326, .556]), on gambling severity, mediated by obsessive passion. All other indirect pathways were non-significant (*p* > .05).

**Table 3.** Indirect pathways between erroneous cognitions, passion, and problem gambling.

Variables	*B*	*S.E.*	*β*	*p*	95% confidence interval for *β*	
					LLCI	ULCI
Illusion of Control ⇒ Harmonious Passion ⇒ Gambling Severity	.007	.010	.015	.456	−.023	.052
**Predictive Control ⇒ Harmonious Passion ⇒ Gambling Severity**	**−.055**	.**014**	**−.145**	**<.001**	**−.216**	**−.073**
Interpretive Bias ⇒ Harmonious Passion ⇒ Gambling Severity	−.016	.011	−.037	.142	−.086	.013
**Illusion of Control ⇒ Obsessive Passion ⇒ Gambling Severity**	.**051**	.**026**	.**102**	.**049**	.**001**	.**204**
Predictive Control ⇒ Obsessive Passion ⇒ Gambling Severity	−.043	.031	−.113	.163	−.269	.043
**Interpretive Bias ⇒ Obsessive Passion ⇒ Gambling Severity**	.**190**	.**029**	.**441**	**<.001**	.**326**	.**556**

Note: *n* = 630. ML estimation with bias-corrected bootstrapping (10,000 resamples). *B* = Unstandardised Beta coefficient, *β* = Standardised Beta coefficient, the strength and direction of the relationship between variables. *S.E*. = Standard Error, *p* = significance value. LLCI = Lower-Level Confidence Interval. ULCI = Upper-Level Confidence Interval. SEM controlled for the shared variance between passion constructs by including the alternative form of passion as a covariate when examining the other. Gambling frequency, social desirability, and strategic and non-strategic gambling were also controlled for. Significant parameters are highlighted in bold text.

### Moderation

Following the SEM and the identification of significant indirect effects, the authors adhered to recommendations from previous research (Edwards & Lambert, 2007; Fairchild & MacKinnon, 2009; Mackinnon, 2011; Preacher et al., 2007) and conducted a post-hoc moderation analysis. This analysis aimed to assess the potential interaction effects among variables. Findings from these moderation analyses are shown in Table 4. All moderation analyses occurred separately, and the effect of the opposing passion construct was controlled for when the other form of passion was added as a moderator.

**Table 4.** Post-hoc moderation analysis.

Model	*B*	*S.E.*	*p*	*β*	95% CI Lower (B)	95% CI Upper (B)
Model 1: Predictive Control						
Predictive Control	−.005	.158	.975	−.001	−.321	.298
Harmonious Passion	**−.931**	.**160**	**<.001**	**−.229**	**−1.238**	**−.614**
Obsessive Passion (Control)	**2.467**	.**136**	**<.001**	.**713**	**2.208**	**2.737**
Gambling Frequency (Control)	.366	.216	.090	.051	−.058	.804
Strategic Gambling (Control)	.**681**	.**204**	.**001**	.**136**	.**285**	**1.089**
Nonstrategic Gambling (Control)	.**887**	.**161**	**<.001**	.**189**	.**578**	**1.211**
Social Desirability	**−1.178**	.**559**	.**035**	**−.058**	**−2.272**	**−.084**
Predictive Control ×Harmonious Passion	−.124	.083	.135	−.044	−.283	.042
Model 2: Illusion of Control						
Illusion of Control	.275	.145	.057	.066	−.001	.565
Obsessive Passion	**2.389**	.**131**	**<.001**	.**690**	**2.138**	**2.650**
Harmonious Passion (Control)	**−1.011**	.**155**	**<.001**	**−.249**	**−1.320**	**−.711**
Gambling Frequency (Control)	.**452**	.**213**	.**034**	.**062**	.**031**	.**877**
Strategic Gambling (Control)	.**633**	.**199**	.**001**	.**127**	.**245**	**1.031**
Nonstrategic Gambling (Control)	.**827**	.**174**	**<.001**	.**176**	.**489**	**1.167**
Social Desirability (Control)	**−1.241**	.**553**	.**025**	**−.061**	**−2.328**	**−.158**
Illusion of Control ×Obsessive Passion	−.046	.131	<.001	0.690	−.179	.083
Model 3: Interpretive bias						
Interpretive bias	.**480**	.**156**	.**002**	.**124**	.**179**	.**792**
Obsessive Passion	**2.388**	.**157**	**<.001**	.**690**	**2.092**	**2.714**
Harmonious Passion (Control)	**−1.084**	.**152**	**<.001**	**−.267**	**−1.382**	**−.789**
Gambling Frequency (Control)	.401	.208	.054	.055	−.015	.815
Strategic Gambling (Control)	.**624**	.**197**	.**002**	.**125**	.**239**	**1.012**
Nonstrategic Gambling (Control)	.**838**	.**163**	**<.001**	.**179**	.**521**	**1.158**
Social Desirability (Control)	−.701	.547	.200	−.034	−1.778	.384
Interpretive Bias ×Obsessive Passion	**−.274**	.**074**	**<.001**	**−.117**	**−.428**	**−.137**

Note: *n* = 630. Bootstrapping (10,000 resamples) was used for this analysis. All predictor and moderator variables were mean-centred prior to creating interactions. This was done to reduce multicollinearity and improve the interpretability of the main effects. Gambling severity scores were treated as raw total scores. B = Unstandardised Beta coefficient, *β* = Standardised Beta coefficient. *S.E*. = Standard Error, *p* = significance value. 95% CI Lower = Lower-Level Confidence Interval. 95% CI Upper = Upper-Level Confidence Interval. Each model controlled for the shared variance between passion constructs by including the alternative form of passion as a covariate when examining the other. Significant parameters are highlighted in bold text. Each moderation model also controlled for gambling frequency, social desirability, and strategic and non-strategic gambling.

Within Model 1, the baseline model accounted for 55.5% of the variance in gambling severity (*R*^2^ = .560, *R*^2^_adjusted_ = .555). However, harmonious passion added no significant variance, Δ*R*^2^ = .002, *F* (1, 621) = 2.49, *p* = .115; *R*^2^_full_ = .561, *R*^2^_full-adjusted_ = .556. Although, obsessive passion remained a significant positive predictor of gambling severity (*β* = .713, *p* < .001), with the control variables of strategic (*β* = .136, *p* = .001) and non-strategic gambling (*β* = .189, *p* < .001) also shown to be positive predictors of gambling severity, whilst social desirability was a significant negative predictor (*β* = −.058, *p* = .035).

In Model 2, 55.7% of the baseline variance (*R*^2^ = .562, *R*^2^_adjusted_ = .557) was explained. Adding the interaction between illusion of control and obsessive passion yielded no significant change (*R*_full_^2^ = .563, *R*^2^_full-adjusted_ = .557; Δ*R*^2^ = .000, *F* (1, 621) = 0.486, *p* = .486). Nevertheless, harmonious passion remained a significant negative predictor of gambling severity within this model (*β* = −.249, *p* < .001), as did gambling frequency (*β* = .062, *p* = .034), as well as strategic (*β* = .127, *p* = .001) and non-strategic gambling (*β* = .189, *p* < .001), which were positive predictors of gambling severity. Social desirability was a significant negative predictor of gambling severity (*β* = −.061, *p* = .025).

In Model 3, when controlling for the effect of harmonious passion, baseline predictors initially accounted for 56.8% of the variance (*R*^2^ = .573, *R*^2^_adjusted_ = .568). Adding the interaction between interpretive bias and obsessive passion significantly increased the explained variance to 57.9% (*R*^2^_full_ = .584, *R*^2^_full-adjusted_ = .579), accounting for an additional 1.2% of unique, incremental variance in gambling severity, Δ*R*^2^ = .012, *F* (1, 621) = 17.41, *p* < .001. Path coefficients revealed obsessive passion as a significant negative moderator of the relationship between interpretive bias and gambling severity (*β* = −.117, *p* < .001). It should also be noted that harmonious passion negatively predicted severity (*β* = −.267, *p* < .001), whilst strategic (*β* = .125, *p* = .002) and non-strategic gambling (*β* = .179, *p* < .001) were positive predictors of gambling severity.

## Discussion

The current study examined the mediating role of harmonious and obsessive passion between several forms of erroneous gambling cognitions (i.e., predictive control, illusion of control and interpretive bias) and gambling severity. Hypotheses regarding direct effects were largely supported, as illusion of control, interpretive bias and obsessive passion positively predicted gambling severity, whereas predictive control and harmonious passion emerged as negative predictors. Predictive control and interpretive bias positively predicted harmonious passion, while illusion of control and interpretive bias positively predicted obsessive passion. Mediation analyses revealed a significant negative partial indirect effect of predictive control on gambling severity via harmonious passion. In contrast, obsessive passion positively mediated the pathways from both illusion of control and interpretive bias to gambling severity. A post hoc moderation analysis was also conducted, with only obsessive passion showing a significant negative interaction effect between interpretive bias and gambling severity.

Notably, participants in the present study reported relatively elevated levels of gambling severity. Given that erroneous gambling cognitions and harmonious/obsessive passion are often more pronounced among individuals experiencing greater gambling problems (Ciccarelli et al., 2016; Leonard et al., 2021; Michalczuk et al., 2011; Morvannou et al., 2017, 2018; Nicholson et al., 2016; Philander & Gainsbury, 2023; Serafim, 2026), the observed structural relationships may be more prominent than in lower-risk problem gambling samples. Consistent with previous literature investigating high (and low) risk gambling samples, erroneous gambling cognitions were significantly positively associated with gambling severity (Ciccarelli et al., 2016; Emond & Marmurek, 2010; Esparza-Reig et al., 2023; Leonard et al., 2021; Nicholson et al., 2016; Philander & Gainsbury, 2023; Schluter et al., 2019). The positive association between obsessive passion and high gambling severity also aligns with prior research (Back et al., 2011; Morvannou et al., 2017, 2018; Philippe & Vallerand, 2007; Serafim, 2026). Although previous research suggests that harmonious passion may be linked with lower gambling severity (Philippe & Vallerand, 2007), current findings add to the mixed literature in this area (Morvannou et al., 2017; Whelan et al., 2021). In particular, whilst zero-order analysis showed a positive link between harmonious passion and gambling severity, partial correlations revealed no significant association once accounting for gambling frequency, gambling preferences, and social desirability, suggesting that the initial correlation may have been underpinned by engagement habits and self-presentation tendencies rather than harmonious passion itself. Furthermore, when obsessive passion and cognitive distortions were integrated into the structural model, harmonious passion emerged as a negative predictor of gambling severity, even though it yielded no independent change in overall explained variance when constrained. This could be due to a suppression effect (i.e., when adding a predictor variable increases the predictive power of another variable; Watson et al., 2013), as examining the incremental variance showed that once obsessive passion was isolated, the total variance increased (as evidenced by the negative Δ*R*^2^). This suggests that the initial zero-order association may have been attributed to shared variance between harmonious and obsessive passion, as well as the potential influence of the control variables. This pattern aligns with findings by Serafim (2026), who found that the positive zero-order correlation between harmonious passion and gambling severity became negative after controlling for obsessive passion, social desirability, and age. This suppression pattern also reflects the conceptualisation of passion within the DMP, indicating that both forms of passion share variance associated with general gambling engagement. Thus, isolating these constructs may better clarify their independent relationships with gambling severity.

Importantly, the positive correlations between harmonious passion and cognitive distortions suggest that gamblers may perceive their gambling as controlled and autonomous while simultaneously endorsing distorted beliefs. Thus, a subjective sense of harmony may not necessarily align with an accurate appraisal of control, nor does it inherently signal lower gambling risk. The positive correlation between predictive control and gambling severity is also consistent with prior research (Awo & Amazue, 2022; Favieri et al., 2025; Flórez et al., 2016; Labrador et al., 2020; Raylu & Oei, 2004). However, the negative predictive effect (i.e., direct effect) between predictive control and gambling severity may be explained by the mediating effect of harmonious passion.

### Mediating role of harmonious passion between predictive control and gambling severity

Harmonious passion mediates the relationship between predictive control and gambling severity, suggesting that greater predictive control was associated with higher harmonious passion, resulting in lower gambling severity. Given the nature of SEM, these pathways may reflect a reciprocal relationship, with low levels of predictive control and harmonious passion leading to higher gambling severity. While the experience of harmonious passion may contribute to an individual’s subjective sense of balance associated with their gambling (Oikonomidis et al., 2019; Vallerand et al., 2003), the structural relationships suggest this positive affect may occur alongside cognitive distortions and negative outcomes. The belief one can influence or predict gambling outcomes may contribute to a sense of agency or mastery that may serve to justify or rationalise continued gambling despite negative outcomes (Clark, 2010; Donati et al., 2018; Favieri et al., 2025; Wu & Clark, 2024; Xian et al., 2008). By overestimating their predictive control over their gambling, individuals may cognitively minimise the problematic nature of their gambling, reframing it as a managed, balanced and/or regulated activity rather than a “problematic” behaviour. As such, individual patterns of responding to the PGSI may be influenced by one’s sense of harmonious passion and lead to an underrepresentation of their degree of gambling severity. This notion aligns with recent empirical research proposing that gamblers who view their gambling as a harmonious activity may struggle and/or fail to recognise their gambling as an issue (Serafim, 2026). Consequently, elevated harmonious passion may function as a cognitive distortion that subverts self-appraisal, fostering an illusory sense of autonomy and behavioural control in spite of the potential adverse consequences of an individual’s gambling.

### Mediating role of obsessive passion between illusion of control and gambling severity

The current study also demonstrated that obsessive passion partially mediates the relationship between illusion of control and gambling severity. As this erroneous cognition reflects an individual’s perceived ability to influence gambling outcomes, this may lead individuals to increase attempts to control their gambling. As an individual increasingly attempts (and fails) to exert control over their gambling outcomes, the failure to achieve the desired outcome may exacerbate their internal compulsion to gamble, thereby intensifying their gambling severity. Therefore, an individual’s erroneous perception of control over gambling outcomes may not act as a direct predictor of higher gambling engagement. Instead, this relationship appears to depend on an individual’s level of obsessive passion towards gambling, which itself may serve as a trait that translates a perceived capacity to influence random events into greater gambling severity. Prominent among behaviours that are related to higher gambling severity is the phenomenon of chasing (i.e., continued gambling often with increasing amounts of money, to offset prior losses; Lesieur, 1979). Individuals demonstrating chasing behaviours may continue to gamble, despite the financial and psychological harms they experience (James et al., 2016; Linnet et al., 2006; Nigro et al., 2018; Sleczka & Romild, 2021). The present findings contribute to the existing literature by indicating that this relationship may be accounted for by an individual’s obsessive passion towards gambling.

### The mediating and moderating role of obsessive passion between interpretive bias and gambling severity

The mediating role of obsessive passion between interpretive bias and gambling severity suggests that the misinterpretation of objective gambling losses acts primarily as a key cognitive vulnerability. Although interpretive bias is recognised as a key variable associated with higher gambling severity (Emond & Marmurek, 2010; Granero et al., 2020; Ledgerwood et al., 2020; Smith et al., 2015), the findings of the present study indicate that this erroneous cognition may also partially rely on intense internal pressures to escalate gambling severity. In this context, an individual’s interpretive bias could fuel a highly repetitive and obsessive drive to gamble until internal beliefs are validated and/or desired outcomes are realised. This is consistent with the underlying conceptualisation of obsessive passion, which posits that this form of passion can inhibit an individual’s behavioural control due to the rigid, uncontrollable urge to gamble (Vallerand, 2010; Vallerand et al., 2003).

Additionally, a post hoc moderation analysis found that obsessive passion, when controlling for harmonious passion, negatively moderated the relationship between interpretive bias and gambling severity. This may indicate that as individuals’ obsession towards their gambling increases, their interpretive bias towards gambling outcomes may become a less salient factor influencing their gambling severity. Thus, higher levels of obsessive passion may lower the explanatory role of erroneous gambling cognitions, with internal pressure potentially emerging as the primary factor associated with higher gambling severity. This idea supports the proposition that obsessive passion, in high-risk gambling samples, may also reflect broader deficits in self-regulation (Serafim, 2026).

### Implications

Findings from the current study have several clinical and theoretical implications. For individuals who subjectively misperceive their gambling as a controlled, harmonious activity, clinicians can utilise personalised normative feedback, a brief intervention that targets cognitive discrepancies (Neighbors et al., 2015). Using personalised normative feedback may assist gamblers in comparing their gambling propensity with normative trends, potentially allowing for the facilitation of more accurate self-appraisals. This comparison may promote a more realistic appraisal of whether their engagement is truly autonomous or fundamentally obsessive.

Clinicians can also use these approaches to help individuals recognise when their sense of control and balance does not match their actual gambling behaviour. Furthermore, as obsessive passion emerged as a more robust factor of gambling severity than interpretive bias, reliance on traditional cognitive restructuring interventions, such as cognitive behavioural therapy (CBT), may be insufficient on their own. While CBT can effectively target erroneous cognitions, findings from the current study highlight that addressing the emotional and motivational drives of gambling (i.e., passion) may also be beneficial. Such approaches could support at-risk gamblers to temper the intense affective states and internal pressures that surround their gambling. With this, as community participants exhibited significantly higher scores across nearly all included variables compared to crowdsourced participants (see supplementary material; https://osf.io/7x6ca), future research should ideally seek to replicate findings in community samples. Targeting community cohorts may be beneficial, as these individuals are often intrinsically motivated to contribute to research (Carr et al., 2022; Carrera et al., 2018; Maund et al., 2020). Conversely, crowdsourced online samples have been shown to be more susceptible to social desirability bias, a pattern observed in the current study, where Prolific participants reported higher social desirability than community participants.

### Limitations and future directions

Several limitations of the current study should be noted. First, while participants were, on average, at moderate risk of problem gambling, researchers did not recruit any clinical (i.e., disordered gamblers). Thereby, findings may be limited to non-clinical demographics. Future researchers may seek to recruit clinical samples and possibly conduct a multi-group SEM, comparing community and clinical gamblers. Second, the sample was predominantly male, limiting generalisability across genders despite higher male gambling rates (Abbott et al., 2018; Carneiro et al., 2020; Wong et al., 2013). Broader gender inclusion may be needed in future studies. Third, as the sample was restricted to adults (18+), findings may not capture developmental nuances in self-regulation and executive function during adolescence (Ferguson et al., 2021; Ma et al., 2026; Meredith & Silvers, 2024). As such, future research should investigate individuals under the age of 18 years or adopt longitudinal designs to clarify these temporal trajectories.

## Conclusion

The current study aimed to explore the roles of obsessive and harmonious passion between erroneous gambling cognitions and gambling severity. By demonstrating that dualistic passion shapes the impact of erroneous beliefs, these findings reveal that underlying motivational drives can modulate and explain how erroneous cognitions influence gambling severity. Notably, when accounting for harmonious passion, obsessive passion emerged as a negative moderator between interpretive bias and gambling severity, indicating that the internal pressure to gamble may overshadow cognitive interpretations of gambling outcomes, potentially becoming a key factor associated with an individual’s gambling severity. This interaction also demonstrates that as a gambling obsession deepens, it may reduce the relative importance of the way in which a gambler interprets the outcomes of their gambling. Future approaches aiming to influence gambling behaviours should consider the role of an individual’s perceptions of gambling outcomes and their motivational passion associated with their gambling.

## Data Availability

The dataset, syntax, consent forms, and advertising materials utilised for this study have been made publicly viewable and available on Open Science Framework (OSF) (see https://osf.io/7x6ca)

## Open scholarship

This article has earned the Center for Open Science badges for Open Data and Open Materials through Open Practices Disclosure. The data and materials are openly accessible at https://osf.io/7x6ca
